# Utility of MRI for Evaluation of a Common Calcaneal Tendon Rupture in a Dog: Case Report

**DOI:** 10.3389/fvets.2020.00602

**Published:** 2020-09-04

**Authors:** Megan Lin, Eric N. Glass, Marc Kent

**Affiliations:** ^1^Section of Neurology and Neurosurgery, Red Bank Veterinary Hospital, Tinton Falls, NJ, United States; ^2^Department of Small Animal Medicine and Surgery, College of Veterinary Medicine, University of Georgia, Athens, GA, United States

**Keywords:** MRI, diagnostic imaging, canine, common calcaneal tendon, tendon lesion

## Abstract

An 8-year-old intact male German shorthaired pointer was presented for a left pelvic limb lameness. Examination revealed a plantigrade stance with flexed digits in the left pelvic limb, and swelling of the left common calcanean tendon distally. Magnetic resonance imaging revealed a partial rupture of the left common calcanean tendon, involving rupture to the tendons of the biceps femoris, gracilis, and semitendinosus muscles. Surgical repair was performed using a modified 3-loop pulley suture. Postoperatively, the tarsus was immobilized with external coaptation. Destabilization of the external coaptation occurred over 9 weeks followed by physical rehabilitation and complete return to function at 10 months post-operative. This case report illustrates the utility of MRI as a diagnostic tool for evaluation of tendon pathology. MRI provided exceptional detail of the tendons that comprise the common calcaneal tendon and the anatomical relationships to surrounding structures which facilitated appropriate surgical correction.

## Background

The common calcanean tendon is composed of the tendons of the superficial digital flexor, gastrocnemius, and the common tendons of the biceps femoris, semitendinosus, and gracilis (1, 2). Although common calcanean tendon (CCT) rupture occurs uncommonly, the cause of CCT rupture typically involves direct trauma (impact or laceration) to the area (3–5). Rupture has also been associated with administration of corticosteroids, fluoroquinolone and non-steroidal anti-inflammatory drugs, obesity, diabetes, and hyperadrenocorticism (1, 4, 6, 7). The dog presented here was a middle-aged athletic hunting dog in which rupture occurred without any obvious trauma. Chronic overuse injury or a degenerative tendon tear are other causes of CCT rupture to consider for the dog of the present report with the signalment and use as a hunting dog (1, 6, 7). Treatment of CCT rupture is surgical repair consisting of either anastomosis of ruptured tendon ends or reattachment of the tendon to the calcaneus in cases of avulsion, post-operative support/immobilization, and rehabilitation (7). CCT ruptures are typically diagnosed based on observation of the gait, posture, and palpation, along with imaging studies including radiographs, ultrasound, and MRI (4). To the authors' knowledge, this is the first case report describing MRI findings of a partial rupture of the left CCT in a dog, and highlights the utility of MRI for evaluation of tendon pathology.

## Case Description

An 8-year-old, 33.6 kg (74-lb) intact male German shorthaired pointer dog was evaluated at Red Bank Veterinary Hospital in Tinton Falls, New Jersey for a 6-week history of a left pelvic limb lameness. The lameness occurred acutely after the dog had been running and the owners heard an audible “pop” sound. The dog was used for hunting, although no known trauma had been witnessed at the time of the onset of the lameness. Initially, the dog was treated by the primary veterinarian with oral minocycline hydrochloride^1^ (2.97 mg/kg [1.35 mg/lb]) by mouth for 28 days. However, the left pelvic limb lameness persisted after 6 weeks and the dog was referred for a possible neurological problem.

Physical examination was normal with the exception of the left pelvic limb. The dog had a grade 2/5 left pelvic limb weight bearing lameness^2^ at a walk. When standing, the dog was plantigrade and the digits of the left pelvic limb were flexed. The distal 3 cm of the left common calcanean tendon was swollen and painful with palpation. The neurological examination was normal.

Based on the physical and neurologic examination findings, the anatomic diagnosis was attributed to a lesion involving the left common calcanean tendon (CCT). The observation of flexed digits while standing suggested that the superficial digital flexor muscle and its tendon remained intact. Differential diagnoses for these signs and history included disruption of either one or more of the components of the CCT, the muscles that contribute to the CCT, or the attachment of the tendon to the calcaneus. Other differentials included a degenerative CCT tear, calcanean tendonitis, or calcaneal fracture.

A complete blood count, serum biochemical profile and 3-view thoracic radiographs were normal. Radiographs of the tarsus were not acquired as the index of suspicion for a calcaneal fracture was low given the soft tissue swelling was primarily located around the left CCT proximal to the calcaneal tuberosity, and combined with the lack of crepitus and inability to palpate subluxation. Under general anesthesia, magnetic resonance imaging (MRI) of the pelvic limbs from the mid-tibia to the mid-metatarsal bones was performed using a 1.5-Tesla unit^3^ and a 15 channel extremity coil. The following multiplanar sequences were obtained with the tarsi in extension: sagittal and anterior-posterior T2-weighted (T2W), sagittal, transverse, and anterior-posterior T1-weighted (T1W), and sagittal short tau inversion recovery (STIR). Following intravenous administration of gadopentetate dimeglumine (0.2 mmol/kg)^4^, sagittal, transverse, and anterior-posterior T1W fat saturated images also were obtained. A vitamin E capsule was used as a marker for all acquisitions.

**Figure 1:**
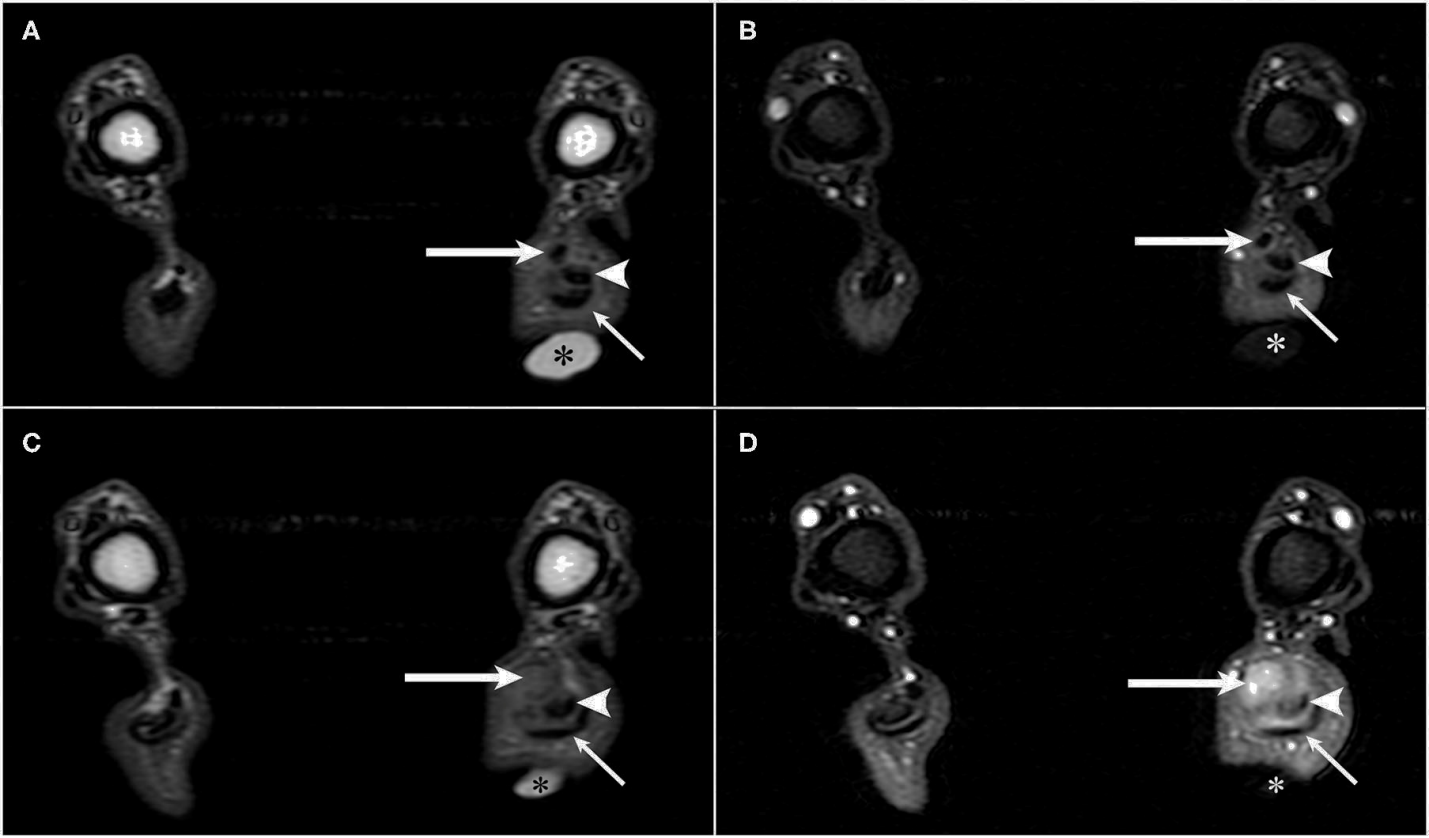
T1-weighted pre- and post-contrast transverse MR images of a German shorthaired pointer dog with rupture of the common tendons of the biceps femoris, gracilis, and semitendinosus (accessory ischial muscles) in the left pelvic limb. A vitamin-E capsule (asterisk) is positioned at the level of the left calcanean tendon visible in images **(A–D)**. **(A)** T1-weighted pre-contrast transverse image of the common tendons of the accessory ischial muscles (long arrow), gastrocnemius tendon (arrowhead), and superficial digital flexor tendon (short arrow). **(B)** T1-weighted post-contrast transverse image of the common tendons of the accessory ischial muscles (long arrow), gastrocnemius tendon (arrowhead), and superficial digital flexor tendon (short arrow) separated by contrast enhancing tissue in the left pelvic limb. **(C)** T1-weighted pre-contrast transverse image showing disruption of the common tendons of the accessory ischial muscles (long arrow) with adjacent gastrocnemius tendon (arrowhead) and superficial digital flexor tendon (short arrow). **(D)** T1-weighted post-contrast transverse image showing disruption of the common tendons of the accessory ischial muscles (long arrow) and contrast enhancement of both the common tendons of the accessory ischial muscles and the gastrocnemius tendon (arrowhead) adjacent to the superficial digital flexor tendon (short arrow).

**Figure 2:**
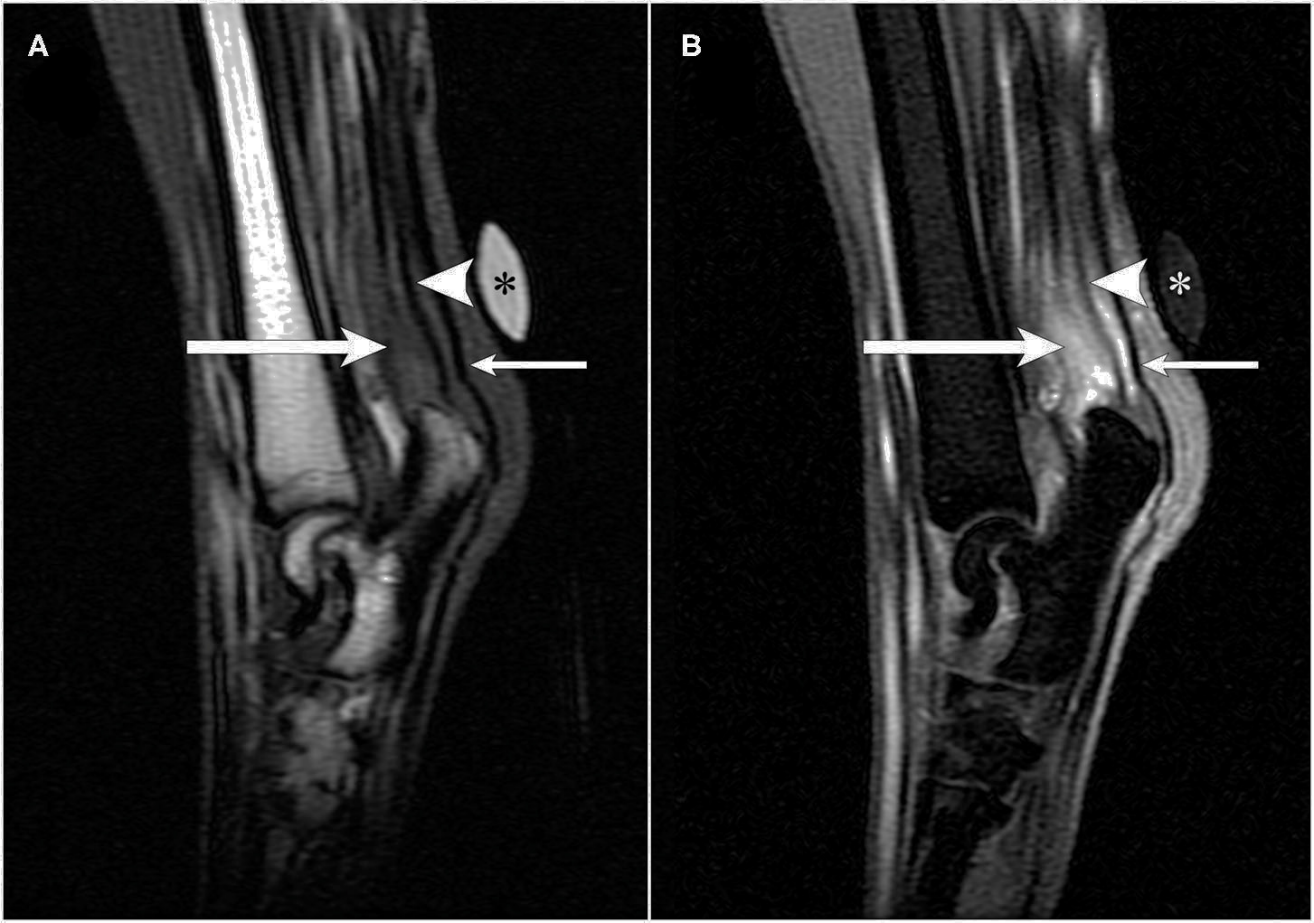
T1-weighted pre- and post-contrast sagittal MR images of a German shorthaired pointer dog with rupture of the common tendons of the biceps femoris, gracilis, and semitendinosus (accessory ischial muscles) in the left pelvic limb. A vitamin-E capsule (asterisk) is positioned at the level of the left calcanean tendon visible in images **(A,B)**. **(A)** T1-weighted pre-contrast sagittal image of the common tendons of the accessory ischial muscles (long arrow), gastrocnemius tendon (arrowhead), and superficial digital flexor tendon (short arrow). The common tendons of the accessory ischial muscles are thickened just proximal to the calcaneus. **(B)** T1-weighted post-contrast sagittal image showing disruption and contrast enhancement of the common tendons of the accessory ischial muscles (long arrow), contrast enhancement surrounding the gastrocnemius tendon with heterogenous signal intensity (arrowhead), and contrast enhancement surrounding the superficial digital flexor tendon (short arrow).

On MRI, ~3-cm proximal to the tuber calcanei, the normal hypointense tendon contributions from the biceps femoris, semitendinosus, and gracilis muscles, also termed as the accessory ischial muscles, abruptly ended (1). From the abrupt end distally, heterogenous T2-hyperintense tissue was present that expanded to ~0.8 cm diameter and resulted in displacement of the fibularis longus muscle cranially and the tendons of the gastrocnemius and superficial digital flexor tendons caudally. This tissue had a heterogeneously, intermediate signal intensity on T1W images, and strongly enhanced (Figure 1). On STIR images, this tissue remained hyperintense. In the sagittal plane, the gastrocnemius and superficial digital flexor tendons were intact; however, the most distal 0.7 cm of the gastrocnemius tendon appeared thinner compared to areas more proximal (Figure 2). Just proximal to this thinner area, there was a 1.0 cm section of the gastrocnemius and superficial digital flexor tendons which were subjectively thickened. It was homogeneously, hypointense on T1W and STIR images and displayed heterogeneous enhancement on T1W post-contrast fat saturated images. Proximal to its attachment to the tuber calcanei, the gastrocnemius tendon also had a focal area of contrast enhancement. Additionally, there was an increased amount of tissue surrounding and dissecting between the gastrocnemius and superficial digital flexor tendons such that they could be easily defined as separate structures as compared to the normal right side. This tissue had heterogeneously intermediate signal intensity on T1W, T2W, and STIR sequences, and displayed heterogenous contrast enhancement. Separate from the tissue dissecting between the gastrocnemius and superficial digital flexor tendons, there was an increased signal intensity surrounding the left gastrocnemius tendon on T2W images and hypointensity on T1W images consistent with edema. This signal intensity was low to intermediate on T1W images. Moreover, the proximal portion of the calcanean bursa, located between the superficial digital flexor tendon and the gastrocnemius tendon, was distended compared to the bursa on the right side, which was normal. The MRI findings were consistent with complete rupture of the tendinous contributions provided by the accessory ischial muscles with presumed tendinitis of the gastrocnemius and superficial digital flexor tendons with concurrent bursitis.

**Figure 3:**
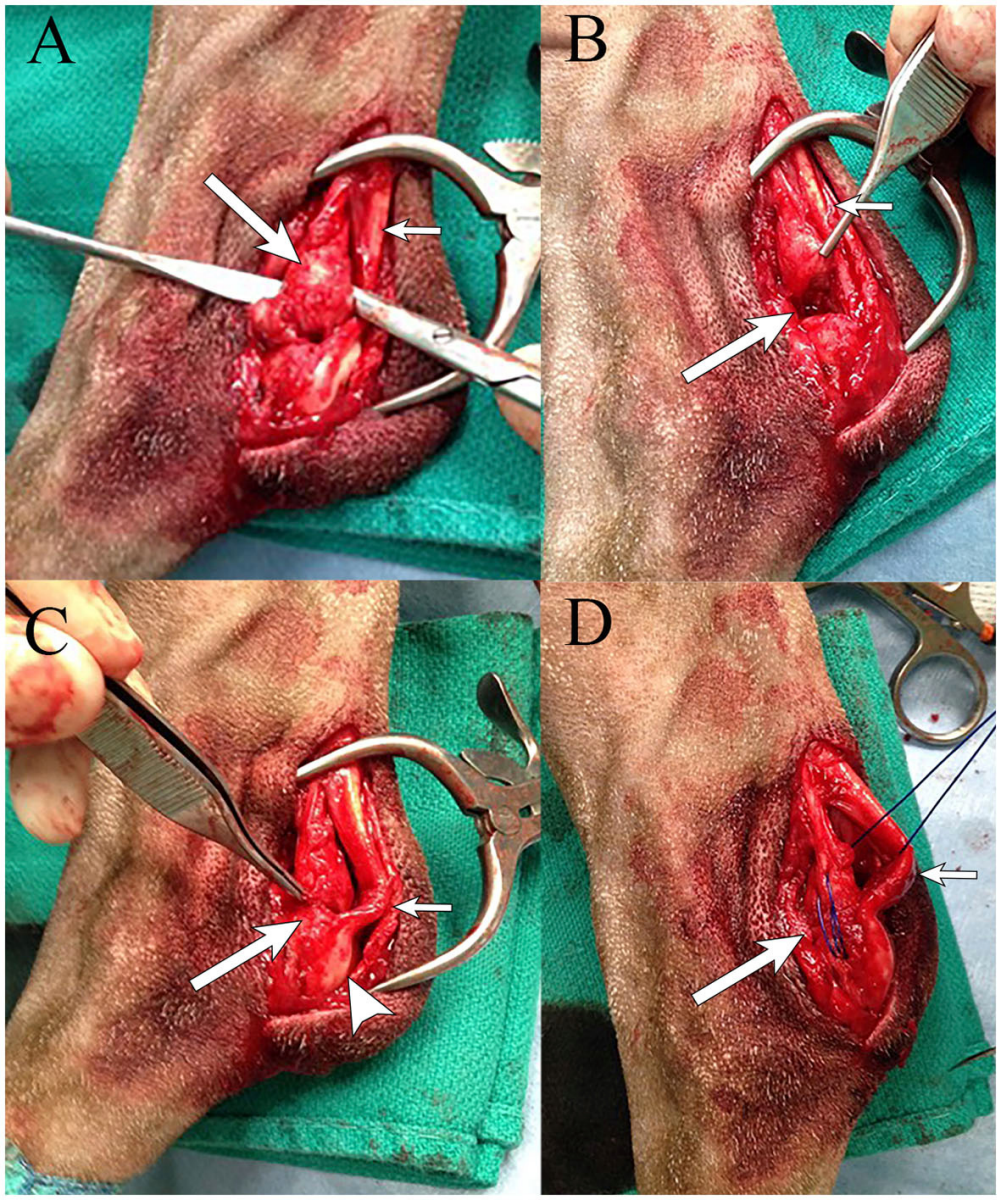
Intraoperative images of a German shorthaired pointer dog with partial rupture of the CCT (rupture of the common tendons of the accessory ischial muscles) in the left pelvic limb. **(A)** Disruption of the common tendons of the accessory ischial muscles (long arrow) next to the gastrocnemius tendon (short arrow). **(B)** Area of excised damaged common tendons of the accessory ischial muscles (long arrow) next to the gastrocnemius tendon (short arrow). **(C)** Reattaching the common tendons of the accessory ischial muscles (long arrow) to the tuber calcaneus (arrowhead), with evidence of a small fold/kink in the gastrocnemius tendon (short arrow). **(D)** Placement of a modified 3-loop pulley suture using #1 prolene (long arrow) in the common tendons of the accessory ischial muscles through the calcaneus, with evidence of a small fold/kink in the gastrocnemius tendon (short arrow).

The dog underwent surgical reattachment of the common tendons of the accessory ischial muscles to the calcaneus. At the level of the tuber calcanei, an ~7 cm skin incision was made over the lateral aspect of the tarsus. A lateral incision was made in the fascial sheath containing the common calcanean tendons. There was a complete disruption of the tendons of the accessory ischial muscles from the tuber calcanei with the distal 2-cm of the tendons having been replaced with fibrous tissue (Figure 3). The gastrocnemius tendon was intact but had palpable thinning close to its attachment to the calcaneus. The superficial digital flexor tendon appeared subjectively normal. The fascia along the lateral edge of the superficial digital flexor tendon was incised and luxated medially. The fibrous tissue as previously described was separated from the adjacent gastrocnemius tendon. Approximately 0.5-cm of the fibrous tissue was debrided. A modified 3-loop pulley suture using a non-absorbable suture^5^ was used to attach the debrided ends of the tendons of the accessory ischial muscles through a 2.0 mm hole drilled through the tuber calcanei. A small fold/kink was identified in the gastrocnemius tendon at its thin portion close to the level of attachment to the calcaneus. A non-absorbable suture^5^ was used to create a reinforcing locking loop suture in the thin region of the distal portion of the gastrocnemius tendon which was anchored using the hole in the tuber calcanei. Using a non-absorbable suture^5^, simple interrupted mattress sutures were placed in the gastrocnemius tendon at the site of its fold. The fascia along the lateral edge of the superficial digital flexor tendon was closed with horizontal mattress sutures using a resorbable suture^6^. The subcutaneous tissue and skin were closed routinely^7^.

Postoperatively, a bivalved cast was applied to the lateral aspect of the left pelvic limb to help protect the repair. The cast extended from just distal to the stifle to the level of the digits and was applied with the tarsus in extension. One day post-operative, the dog was discharged on tramadol^8^ [3 mg/kg *per os* [PO] *q* 8–12 hr], and deracoxib^9^ (1.5 mg/kg PO *q* 24 hr) for 7 days and instructions for restricted activity for 3 months. In an effort to gradually allow weight bearing forces to be applied to the tendon, destabilization of the external coaptation occurred over the course of 9-weeks following surgery. Objective evaluation of the load applied to the CCT was not performed. Destabilization was subjectively felt accomplished by the use of the bivalved cast for 6-weeks, followed by a lateral splint for 2-weeks, and then a soft padded wrap for 1-week. Bandage changes were performed every 2-weeks. At each bandage change, confirmation of the integrity of the surgical attachment of the tendons was evidenced by the inability to flex the tarsus while the stifle was held in extension. Following the 9-weeks of external coaptation, a nylon Thera-paw^10^ custom tarsal support wrap with straps fitting around the hock and inner foam padding was used for an additional 5-weeks.

Nine weeks post-operatively, the dog was started on a physical rehabilitation therapy program. For an 8-month period (with therapy sessions ranging from once weekly to once every 2 weeks for a total of 15 sessions), the dog received treatments which included passive range of motion, underwater treadmill walking, massage, low level laser therapy, and thermotherapy. By 9 months post-operatively, there was absence of lameness based on examination by a diplomate of the American College of Veterinary Sports Medicine and Rehabilitation. Ten months post-operatively, the dog had recovered well-enough to resume his previous hunting activity.

## Discussion

Imaging studies used in the diagnosis of CCT rupture include radiographs, ultrasound, and MRI. This case highlights the utility of MRI in several ways. MRI provides the best soft tissue contrast compared with all imaging modalities and therefore is anticipated to be sensitive for identifying pathologic processes involving tendinous structures. In addition, high resolution, multiplanar images can be acquired to facilitate the assessment of both soft tissue and bone pathology (8). In the case herein, areas of signal change within the tendons likely signified additional pathologies such as edema and changes in tendon thickness which were easily discerned. Adding to this exceptional soft tissue contrast resolution provided by MRI, the T2-hyperintensity combined with T1 hypointensity consistent with the edema in the tissues surrounding the tendons helped further delineate the individual tendinous contributions of the CCT in the present case as normal tendons are hypointense on most sequences.

Another advantage of MRI over other imaging modalities such as ultrasonography is that images can be acquired which enable broad visualization of surrounding structures in one single image. This allows assessment of the anatomical relationships between the tendons and surrounding structures such as bone, ligaments, and muscle. Additionally, gradient echo sequences could be used to evaluate for hemorrhage in acute CCT injuries, and proton density weighted (PDW) images with fat saturation also are useful in evaluation of bony structures such as the ridges of the talus, and for identification of ligaments (8). Finally, MRI may help identify pathology in the subchondral bone at the site of tendon insertions as well as intraarticular lesions (9). Conversely, there also are several important limitations to consider with MRI compared to ultrasonography including cost and the need for general anesthesia. While possible with MRI, the acquisition of images with the tarsus in varying positions is more readily performed with ultrasonography (10). Similarly, intraoperative ultrasonography can be used during surgical repair to help identify structures correctly. Specific limitations in the present report includes the lack of PDW images and lack of repeated imaging over time. Proton density weighted sequences provide for a detailed assessment of tendons surrounded by fluid such as the gastrocnemius tendon (8). This may have been useful in the dog of the present report given the increased T2W signal intensity that surrounded the left gastrocnemius tendon and increased T1W signal intensity of the left gastrocnemius tendon.

Ultrasonography has a high diagnostic accuracy for tendinopathies (11). In veterinary medicine, ultrasonography is the primary imaging modality used for the evaluation of tendinopathies. In humans, ultrasonography may not be sufficiently reliable for the diagnosis of all CCT pathologies including partial CCT ruptures (12). Despite this, ultrasonography in veterinary medicine remains a valuable diagnostic imaging modality as it is readily available in most practices, does not necessitate the animal being under general anesthesia to acquire images, and has a lesser cost compared to MRI. Such advantages allow for performance of repeated studies over time. Importantly, ultrasonographic abnormalities do correlate with observations made at surgery (13). Disadvantages of ultrasonography include the need for expertise by the imager, quality of the ultrasound unit, and the need for an acoustic window for evaluation of some ligamentous structures (10). Despite these disadvantages, ultrasonography should be considered a first-line imaging modality used to localize tendon injuries and characterize different grades of CCT injury (4, 13–15). In the end, MRI may complement ultrasonography given its ability to provide greatly detailed images of tendon pathology (12). Consequently, MRI can be useful in animals when CCT rupture is suspected, or in cases in which radiographs or ultrasound fail to identify a clear cause for persistent lameness in an animal with suspected CCT rupture (8).

Recommended MRI sequences for evaluating the tarsal region should include T1W pre- and post-contrast, T1W with fat saturation, T2W, T2W with fat saturation, STIR (short tau inversion recovery), GRE (gradient echo), FLAIR (fluid attenuated inversion recovery), and PDW (8, 16). Common musculoskeletal pathology seen on MRI includes inflammation, neoplasia, fibrosis, fatty infiltration, and hemorrhage (8). Edema consequent to inflammation or trauma can be appreciated on T2W images as hyperintense anatomy compared to normal tissue, and as low signal structures on T1W images (8, 17). Such signal changes characteristics of edema were seen surrounding the gastrocnemius tendon and superficial digital flexor tendon on the T2W images of the dog in this report. The use of intravenous contrast for tendon imaging also can help to identify abnormal tissue, tendon tears, inflammation, and neoplasia (8, 10, 16). Additional findings with tendon abnormalities include thickening or thinning of the tendon, and high T2 signal intensity surrounding the tendon and abnormal contrast-enhancement (10). T1W images of the dog of the present report revealed focal thickening of the gastrocnemius and superficial digital flexor tendons as well as a 0.7 cm long focal thinning of the gastrocnemius tendon at the tendo-osseous junction. However, MRI did not enable identification on the small kink of the gastrocnemius tendon close to its attachment to the calcaneus which was only seen intra-operatively. These findings may have been a consequence of positioning the tarsus in extension for the MRI.

As in the present case, information gained through MRI should better serve to guide treatment recommendations and surgical repair over data provided by other imaging modalities. In acute cases of CCT injury, it is recommended to suture each component of the CCT individually to reestablish tendon integrity (1). In the present case, the ability of MRI to show that the gastrocnemius tendon and superficial digital flexor tendon were intact but had pathology helped surgeon expectations prior to surgical exploration, guided tissue handling, and potentially aided in the prognosis of the repair. Further research is needed to elucidate whether MRI specific findings of tendon pathology may help determine or necessitate different types of tendon repair. For example, other repair methods include using a semitendinosus muscle flap in addition to a three-loop pulley pattern, use of a plate, fascia lata graft, or transposition of the peroneus brevis muscle/tendon, peroneus longus tendon, or deep digital flexor tendon (18–21). To date, CCT repair outcomes do not vary with suture pattern chosen for tendon anastomosis as long as the immobilization method post-operative is successful; typically complications relate to the immobilization method rather than the surgical repair (22, 23). Lastly, MRI should enable discrimination of other conditions that result in clinical signs similar to CCT rupture. Disruption of the long plantar ligament also results in a plantigrade posture and digit hyperflexion (24, 25). If a long plantar ligament rupture is diagnosed instead of a CCT rupture, the appropriate surgical treatment is arthrodesis (22, 25). In such instances, MRI may prove useful for an accurate diagnosis of a long plantar ligament rupture and choice of surgical repair.

## Concluding Remarks

To the authors' knowledge, this is the first case report describing MRI findings of a partial rupture of the left CCT in a dog. Although MRI may not be feasible in every veterinary patient, use of MRI to evaluate CCT rupture and other musculoskeletal diseases should be recommended to clients and considered as a valuable diagnostic tool. Further research regarding the benefits of MRI for evaluating CCT rupture and tendon pathology should be pursued to improve diagnosis and treatment of affected patients.

## Data Availability Statement

The original contributions presented in the study are included in the article/Supplementary Material, further inquiries can be directed to the corresponding author/s.

## Ethics Statement

Written informed consent was obtained from the owners for the participation of their animals in this study.

## Author Contributions

ML contributed to writing the manuscript, interpreting and describing the imaging findings, and literature review. EG was the main clinician during case presentation and performed the neurologic consultation. EG and MK contributed to writing and editing the manuscript and interpreting and describing the imaging findings. All authors contributed to the article and approved the submitted version.

## Conflict of Interest

The authors declare that the research was conducted in the absence of any commercial or financial relationships that could be construed as a potential conflict of interest.

## Footnotes

^1^*Minocycline Hydrochloride*. Parsippany, NJ: Watson Pharmaceuticals, Inc.

^2^Fossum TW. *Box 11-2, Lameness Scoring. Principals of Physical Rehabilitation. Small Animal Surgery*. St. Louis, MO: Elsevier Mosby (2013). p. 125.

^3^*GE Brivo MR 355 Inspire*. Princeton, NJ: GE Healthcare.

^4^*Magnevist (Gadopentetate Dimeglumine)*. Wayne, NJ: Bayer Healthcare Pharmaceuticals.

^5^*Prolene (Polypropylene) Suture*. Somerville: NJ: Ethicon Inc.

^6^*PDS II (Polydioxanone) Suture*. Juarez: Ethicon Inc.,

^7^*Monocryl (Poliglecaprone 25) Suture*. Cornelia: Ethicon Inc.

^8^*Tramadol Hydrochloride*. Hauppauge, NY: Amneal Pharmaceuticals.

^9^*Deramaxx*. Greenfield: Elanco US Inc.

^10^*Thera-Paw*. Lebanon, NJ: Thera-Paw, Inc.
