# Supplementary material for: Utility of MRI for Evaluation of a Common Calcaneal Tendon Rupture in a Dog: Case Report
Source: Front Vet Sci. 2020 Sep 4;7:602. doi: 10.3389/fvets.2020.00602 (PMC7498657; doi:10.3389/fvets.2020.00602)
Supplement: Supplementary file 1 [file Data_Sheet_1.PDF]

CARE
case report guidelines

CARE Checklist of information to include when writing a case report

(cc) BY-NC-ND

Topic	Item	Checklist item description	Reported on Line
Title	1	The diagnosis or intervention of primary focus followed by the words "case report"	3
Key Words	2	2 to 5 key words that identify diagnoses or interventions in this case report, including "case report"	15
Abstract (no references)	3a	Introduction: What is unique about this case and what does it add to the scientific literature?	28-31
	3b	Main symptoms and/or important clinical findings	21-25
	3c	The main diagnoses, therapeutic interventions, and outcomes	23-28
	3d	Conclusion—What is the main "take-away" lesson(s) from this case?	28-31
Introduction	4	One or two paragraphs summarizing why this case is unique (**may include references**)	40-41, 47-49
Patient Information	5a	De-identified patient specific information.	51
	5b	Primary concerns and symptoms of the patient.	51-58
	5c	Medical, family, and psycho-social history including relevant genetic information	n/a
	5d	Relevant past interventions with outcomes	55-57
Clinical Findings	6	Describe significant physical examination (PE) and important clinical findings.	59-63
Timeline	7	Historical and current information from this episode of care organized as a timeline	51-78
Diagnostic Assessment	8a	Diagnostic testing (such as PE, laboratory testing, imaging, surveys).	73-78
	8b	Diagnostic challenges (such as access to testing, financial, or cultural)	n/a
	8c	Diagnosis (including other diagnoses considered)	79-99
	8d	Prognosis (such as staging in oncology) where applicable	n/a
Therapeutic Intervention	9a	Types of therapeutic intervention (such as pharmacologic, surgical, preventive, self-care)	100-139
	9b	Administration of therapeutic intervention (such as dosage, strength, duration)	120-139
	9c	Changes in therapeutic intervention (with rationale)	120-139
Follow-up and Outcomes	10a	Clinician and patient-assessed outcomes (if available)	132-139
	10b	Important follow-up diagnostic and other test results	132-139
	10c	Intervention adherence and tolerability (How was this assessed?)	n/a
	10d	Adverse and unanticipated events	n/a
Discussion	11a	A scientific discussion of the strengths AND limitations associated with this case report	186–198
	11b	Discussion of the relevant medical literature **with references**.	141-210
	11c	The scientific rationale for any conclusions (including assessment of possible causes)	141-210
	11d	The primary "take-away" lessons of this case report (without references) in a one paragraph conclusion	212-217
Patient Perspective	12	The patient should share their perspective in one to two paragraphs on the treatment(s) they received	n/a
Informed Consent	13	Did the patient give informed consent? Please provide if requested	n/a Yes ☐ No ☐
